# Statistical data pre-processing and time series incorporation for high-efficacy calibration of low-cost NO_2_ sensor using machine learning

**DOI:** 10.1038/s41598-024-59993-6

**Published:** 2024-04-21

**Authors:** Slawomir Koziel, Anna Pietrenko-Dabrowska, Marek Wojcikowski, Bogdan Pankiewicz

**Affiliations:** 1 Engineering Optimization and Modeling Center, Reykjavik University, 102 Reykjavik, Iceland; 2 Faculty of Electronics, Telecommunications and Informatics, Gdansk University of Technology, 80-233 Gdansk, Poland

**Keywords:** Air pollution, Low-cost sensor calibration, Machine learning, Data pre-processing, Neural networks, Environmental impact, Electrical and electronic engineering, Atmospheric science

## Abstract

Air pollution stands as a significant modern-day challenge impacting life quality, the environment, and the economy. It comprises various pollutants like gases, particulate matter, biological molecules, and more, stemming from sources such as vehicle emissions, industrial operations, agriculture, and natural events. Nitrogen dioxide (NO_2_), among these harmful gases, is notably prevalent in densely populated urban regions. Given its adverse effects on health and the environment, accurate monitoring of NO_2_ levels becomes imperative for devising effective risk mitigation strategies. However, the precise measurement of NO_2_ poses challenges as it traditionally relies on costly and bulky equipment. This has prompted the development of more affordable alternatives, although their reliability is often questionable. The aim of this article is to introduce a groundbreaking method for precisely calibrating cost-effective NO_2_ sensors. This technique involves statistical preprocessing of low-cost sensor readings, aligning their distribution with reference data. Central to this calibration is an artificial neural network (ANN) surrogate designed to predict sensor correction coefficients. It utilizes environmental variables (temperature, humidity, atmospheric pressure), cross-references auxiliary NO_2_ sensors, and incorporates short time series of previous readings from the primary sensor. These methods are complemented by global data scaling. Demonstrated using a custom-designed cost-effective monitoring platform and high-precision public reference station data collected over 5 months, every component of our calibration framework proves crucial, contributing to its exceptional accuracy (with a correlation coefficient near 0.95 concerning the reference data and an RMSE below 2.4 µg/m^3^). This level of performance positions the calibrated sensor as a viable, cost-effective alternative to traditional monitoring approaches.

## Introduction

Nitrogen dioxide (NO_2_) pollution is a significant environmental concern stemming from various sources such as vehicle emissions, industrial processes, and combustion. This gas is a part of nitrogen oxides (NO_x_) and contributes to poor air quality, leading to respiratory issues and environmental damage. NO_2_ reacts in the atmosphere to form harmful particles and ozone, impacting human health, ecosystems, and even contributing to climate change^1–6^. Specifically, emissions of NO_x_ play a key role in creating photochemical smog, triggering acid rain, and causing ecological harm in water reservoirs^7^. Furthermore, high NO_x_ levels also elevate O_3_, adversely affecting agriculture. Needless to say, monitoring and reducing NO_2_ levels are critical for mitigating its adverse effects on both human health and the environment. Strict regulations have been implemented to control NO_2_ levels, such as the CAFE Directive, setting an annual average below 40 µg/m^3^ and hourly concentrations not exceeding 200 µg/m^3^ for over 18 h per year^8^. The World Health Organization (WHO) has proposed even stricter limits^9^. However, about one-sixth of European monitoring stations indicate NO_2_ levels that surpass these boundaries, especially in urban zones, particularly along transportation corridors. The economic toll of air pollution, including NO_2_, amounts to significant costs^2,10^.

Traditional methods for NO_2_ monitoring rely on stationary and bulky equipment, demanding controlled environments and regular maintenance. Commonly used measurement approaches encompass photofragment chemiluminescence^11^, long-range differential optical absorption spectroscopy^12^, laser-induced fluorescence^13^, and cavity ring down spectroscopy^14^. Although these methods exhibit high sensitivity, some present limitations (e.g. unsuitability for localized monitoring^12^) or require intricate hardware (e.g., a vacuum system and a pulsed laser^13^). These deficiencies in traditional monitoring systems have driven the development of alternative methods that are cost-effective, easily deployable, and straightforward to maintain. In recent years, considerable research efforts have been directed towards development of portable platforms, which may be useful to enhance the spatial resolution of air quality monitoring. The latter is essential for urban areas with diverse pollutant distributions^15–17^. Notwithstanding, low-cost sensors encounter reliability limitations^18–20^ due to instability^21^, fabrication inaccuracies^22,23^, and cross-sensitivity to multiple gases^24–26^. They are also sensitive to environmental conditions, especially temperature and humidity^27,28^. In spite of these constraints, affordable sensors may complement sparsely positioned reference stations and serve as cost-efficient air quality monitoring solutions^29^. They may also become foundations of integrated sensor networks^30,31^, including those deployed on cars or aerial vehicles^32,33^.

Enhancing the reliability of low-cost sensors has been a focal point in research, primarily focusing on refining calibration methods. These techniques are typically categorized into two types: laboratory-based and field-based^34^. While laboratory procedures are more precise in theory, they often fall short in practice as the actual operating conditions of sensors seldom align with controlled laboratory settings^18,19^. Consequently, field-based techniques are more prevalent, relying on reference data collected from public air monitoring stations. Numerical modelling for calibration typically involves either rudimentary regression techniques or more advanced machine learning approaches. In Ref.^35^, methods such as multivariate linear regression (MLR), support vector regression (SVR), and random forest regression (RFR) were employed to calibrate electrochemical NO and NO_2_ sensors based on temperature and humidity data. A study presented in Ref.^36^ utilized ridge regression, random forest regression (RFR), Gaussian process regression (GPR), and MLR to correct low-cost NO_2_ and PM_10_ sensors based on temperature and humidity. In Ref.^37^, calibration of a chemiluminescence NO-NO_2_-NO_x_ analyser using MLR was showcased, also integrating temperature and humidity data. Further investigations into diverse regression models have been reported in Refs.^38–40^.

In recent times, there has been a surge in interest in employing artificial intelligence methods, specifically neural networks (NNs) and diverse machine learning techniques, to achieve more dependable correction of low-cost sensors. For instance, Ref.^29^ employed single linear regression (SLR), multivariate linear regression (MLR), random forest regression (RFR), and long short-term memory networks (LSTM) for calibrating CO, NO_2_, O_3_, and SO_2_ sensors, noting LSTM's superior performance compared to regression procedures. Meanwhile, in Ref.^15^, convolutional neural networks (CNNs) and recurrent neural networks (RNNs) were used to calibrate CO and O_3_ sensors using temperature and humidity data, showcasing advantages over linear regression (LR), SVR, or LSTM combined with CNN. Extensive literature, as observed in Refs.^41–44^, showcases the application of various ANN surrogates, e.g. Bayesian NNs, shallow NNs, or dynamic NNs for low-cost sensor calibration.

In this research, we introduce an innovative method for precise calibration of affordable NO_2_ sensors. The technique revolves around statistical preprocessing of low-cost sensor data to align its distribution with reference data before further refinement. Central to this approach is an artificial neural network (ANN) surrogate, tailored to predict sensor correction coefficients that encompass additive adjustment and multiplicative scaling. The surrogate model is trained using environmental variables (temperature, humidity, atmospheric pressure), data cross-referenced from auxiliary NO_2_ sensors, and short time series of previous readings from the primary sensor. Global data scaling is also integrated as an additional calibration mechanism. To validate our calibration methodology, we applied it to a custom-designed autonomous monitoring platform equipped with NO_2_ and environmental detectors, supported by electronic circuitry for monitoring implementation and data transfer protocols. Reference data was collected over five months from high-precision public stations in Gdansk, Poland. The results demonstrate exceptional calibration efficacy, achieving a correlation coefficient close to 0.95 with reference data and an extremely low RMSE below 2.4 µg/m^3^, even within a broad NO_2_ measurement range (from zero to sixty µg/m^3^). Additional experiments conducted with different sets of surrogate model inputs and by excluding certain algorithmic tools highlight the vital role of each mechanism within the calibration framework, reaffirming their significance in enhancing correction quality.

## Autonomous NO_2_ monitoring platform

The article will showcase the sensor calibration methodology implemented on a custom-designed autonomous monitoring platform developed at Gdansk University of Technology, Poland. Section "Hardware description" details the hardware specifications, while Section "Monitoring platform: output data" delves into the data output from the platform's sensors.

### Hardware description

The system is a comprehensive setup comprising multiple sensors for monitoring environmental factors such as temperature, humidity, and atmospheric pressure. It integrates a primary nitrogen dioxide sensor and two redundant sensors for cross-validation purposes. Furthermore, it includes a GSM modem for wirelessly transmitting measurement data to the cloud. Managing the air quality monitoring protocols are off-the-shelf components coordinated by the BeagleBone^®^ Blue microprocessor system^45^, which houses a 1 GHz ARM^®^ Cortex-A8 processor, 512 MB DDR3 RAM, and 4 GB eMMC memory, operating on the Linux OS.

**Figure 1:**
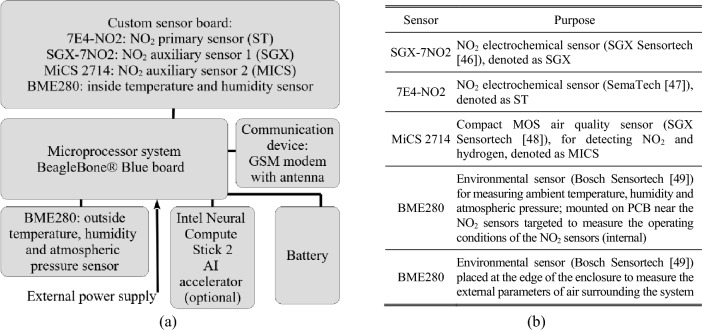
Autonomous air monitoring platform designed at Gdansk University of Technology, Poland: (**a**) block diagram, (**b**) included sensors^46–49^.

**Figure 2:**
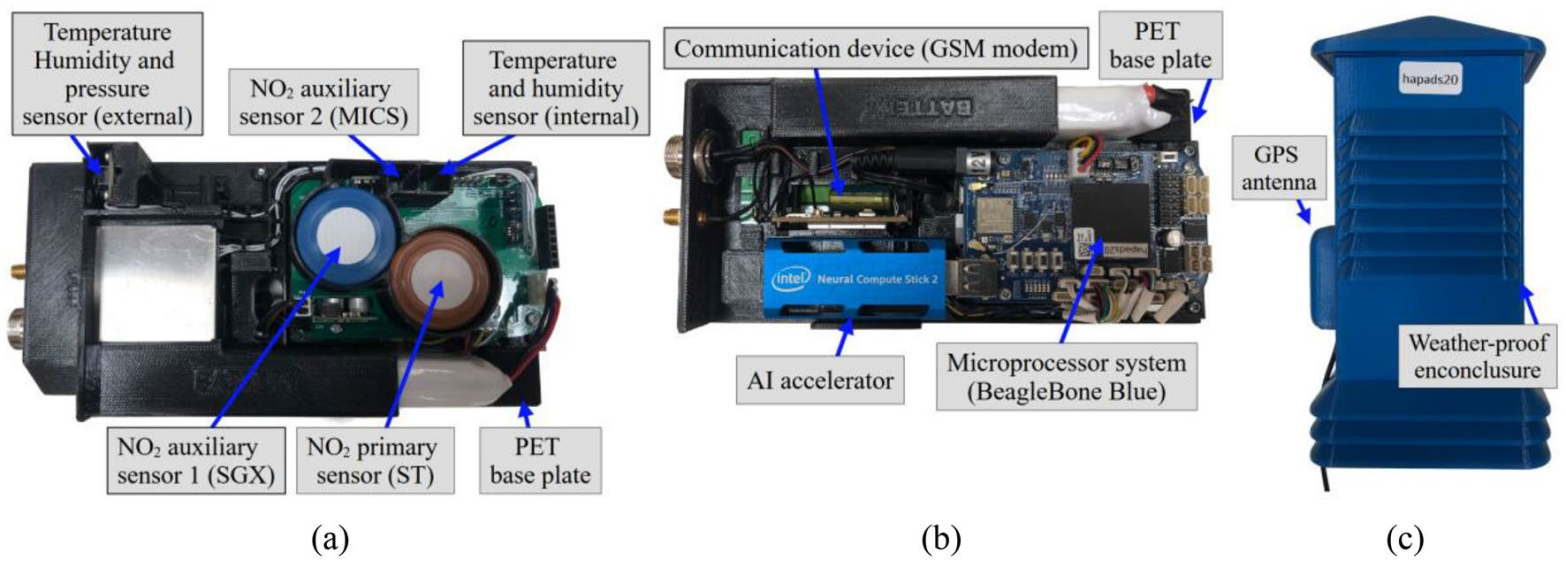
Autonomous monitoring platform designed at Gdansk University of Technology, Poland: (**a**) internals (top view), (**b**) internals (bottom view), (**c**) systems mounted in weather-proof enclosure.

The system relies on a rechargeable 7.4 V/4400 mA battery capable of sustaining operations for at least twenty hours without external power sources. The block diagram of the platform, featuring sensor details, is illustrated in Fig. 1. Data transmission occurs via the GSM modem, making the measurement data available online. The system is mounted on a polyethylene terephthalate base plate, as depicted in Fig. 2. The gas sensors (ST, SGX, MICS) are closely positioned (see Fig. 2a) along with environmental detectors monitoring their operational conditions. An auxiliary environmental sensor is placed at the device's edge.

The employment of auxiliary sensors serves to address variations between external and internal temperatures and humidity, primarily influenced by heat generated by the electronic circuitry. An Intel USB Stick module is also installed for potential on-board execution of calibration procedures. The platform is accommodated in a weatherproof enclosure, cf. Fig. 2c.

### Monitoring platform: output data

**Figure 3:**
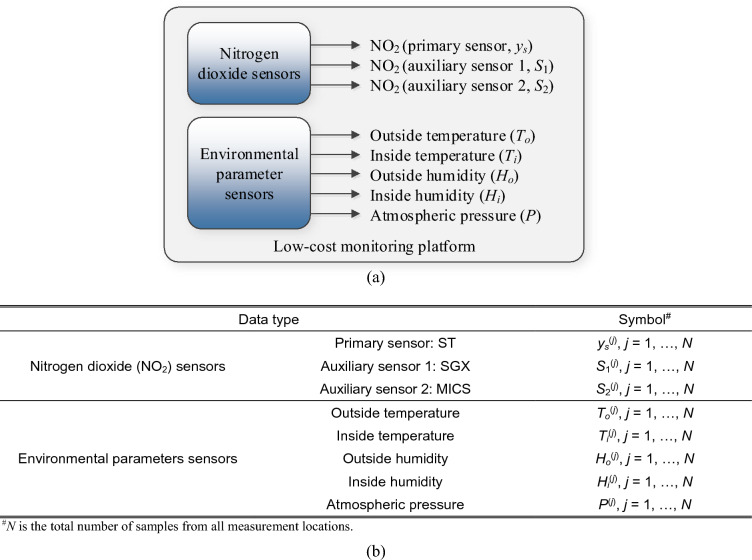
Outputs of the low-cost monitoring platform of Section "Hardware description": (**a**) NO_2_ reading from the low-cost sensor under calibration (*y*_*s*_). The sensor also produces auxiliary outputs: auxiliary NO_2_ readings (*S*_1_ and *S*_2_), outside and inside temperature (*T*_*o*_ and *T*_*i*_, respectively), outside and inside humidity (*H*_*o*_ and *H*_*i*_, respectively), and atmospheric pressure (*P*); (**b**) symbols of data produced by the platform’s sensors. The number *N* stands for the total number of data samples obtained from the platforms, further divided into training and testing sets (cf. Section "Precise sensor calibration using statistical pre-processing, ANN surrogates, and global data scaling").

The monitoring platform, detailed in Section "Hardware description", gathers NO_2_ measurements from the primary sensor and two redundant sensors, along with environmental sensor data (internal and external temperature, humidity, and atmospheric pressure). Figure 3a visually represents these outputs, while Fig. 3b introduces the notation used in this study. It is crucial to note that this platform captures environmental parameters both within the system (close to the NO_2_ sensors) and externally (at the edge of the platform). The variations in internal and external temperature and humidity stem from the heat produced by the electronic circuitry. Given the influence of these parameters on sensor performance, incorporating both sets of temperature and humidity data can significantly enhance the reliability of the calibration process. Additionally, although the accuracy of the auxiliary NO_2_ sensors within the platform is limited, their readings offer indirect yet valuable insights into the factors affecting the primary sensor, notably its cross-sensitivity to other gases.

## Reference data. Public monitoring stations

**Figure 4:**
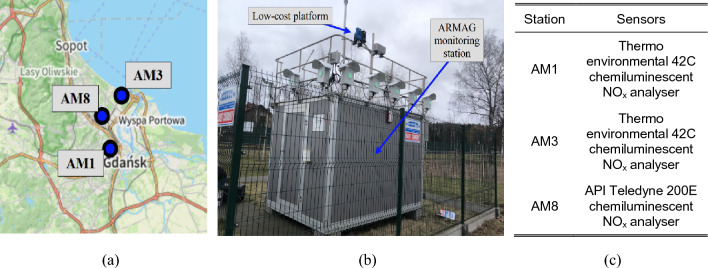
Reference monitoring stations of the ARMAG foundation used to acquire reference data: (**a**) station locations in the city of Gdansk, (**b**) photograph of the selected station with the proposed low-cost platform mounted in the vicinity, (**c**) NO_x_ sensors installed on the stations.

The calibration process for the low-cost sensor will utilize reference data obtained from high-precision public monitoring stations strategically located in Gdansk, Poland, operated by the ARMAG Foundation^50^. The geographical distribution of these stations is illustrated in Fig. 4a. The stations are housed within air-conditioned containers and are equipped with high-performance air monitoring instruments, detailed in Fig. 4b. The specific sensors used for NO-NO_2_-NO_x_ measurements are listed in Fig. 4c. ARMAG provides open access to the generated data on their website (https://armaag.gda.pl/en/). Measurements are carried out hourly and are accessible on the foundation’s website for a duration of three days. To enable extended data collection periods, a custom script has been prepared, which allows automated download of this information into a text file hosted on a dedicated server.

## Precise sensor calibration using statistical pre-processing, ANN surrogates, and global data scaling

This section delineates the comprehensive methodology devised for the calibration of low-cost NO_2_ sensors. The task of correcting the sensor is formulated in Section "Sensor calibration. Problem statement". Further details regarding the affine correction scheme are provided in Section "Additive and multiplicative low-cost sensor correction". Section "Statistical pre-processing of low-cost sensor measurements" delves into the statistical pre-processing of data, designed to enhance the initial alignment between the outputs of the reference and low-cost sensors. An in-depth exploration of the primary calibration model, an artificial neural network (ANN) surrogate, is presented in Section "Sensor calibration using neural network surrogate". The various configurations of inputs to the ANN model are elucidated in Section "Calibration model inputs". These encompass fundamental environmental parameters and redundant NO_2_ sensor readings (Section "Calibration input configuration I: basic setup"), expanded sets incorporating differentials (Section "Calibration input configuration II: differentials"), and time-series-based inputs comprising prior NO_2_ measurements from the primary sensor (Section "Calibration input configuration III: time series of prior NO2 measurements"). Additionally, Section "Global data scaling" discusses an auxiliary calibration mechanism, specifically global data scaling. The comprehensive workflow for NO_2_ monitoring utilizing the calibrated low-cost sensor is elucidated in Section "Operating flow of NO2 monitoring by means of calibrated sensor".

### Sensor calibration. Problem statement

**Figure 5:**
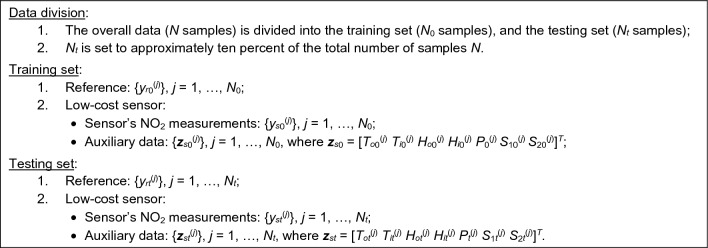
Division of the reference and low-cost sensor data into training and testing set.

Sensor calibration is based on two datasets. The first one comprises NO_2_ readings obtained from the reference stations, as outlined in Section "Reference data. Public monitoring stations". The respective samples will be denoted as *y*_*r*_^(*j*)^, *j* = 1, …, *N*, where *N* is the total number of points. The datasets obtained from the autonomous platform described in Section "Autonomous NO2 monitoring platform", i.e., {*y*_*s*_^(*j*)^} and the respective environmental parameter vectors {***z***_*s*_^(*j*)^} (cf. Fig. 3) is in correspondence with {*y*_*r*_^(*j*)^}, i.e., the respective outputs are collected at the same time intervals. Figure 5 elucidates the division of this data into training and testing sets. The testing set consists of several two-week sequences gathered at different time intervals during the five-month measurement campaign, as elaborated in Section "Results and discussion".

**Figure 6:**
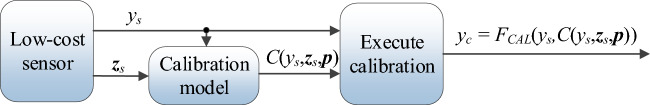
Overall flow of the low-cost sensor calibration. Auxiliary data and sensor output *y*_*s*_ are used to obtain the correction coefficients *C*(*y*_*s*_,***z***_*s*_,***p***), which are then used to compute the corrected sensor output *y*_*c*_, see Sections "Additive and multiplicative low-cost sensor correction" through Sect. "Global data scaling" for details. A more detailed procedure will be discussed in Section "Global data scaling".

Sensor calibration is realized using the training datasets {*y*_*r*0_^(*j*)^}, {*y*_*s*0_^(*j*)^}, and {***z***_*s*0_^(*j*)^}, *j* = 1, …, *N*_0_ (cf. Fig. 5). The correction coefficients are jointly denoted as *C*(*y*_*s*_,***z***_*s*_;***p***), cf. Fig. 6, where ***p*** stands for the combined calibration model hyper-parameters. The corrected sensor’s output is denoted as *y*_*c*_ = *F*_*CAL*_(*y*_*s*_,*C*(*y*_*s*_,***z***_*s*_;***p***)). Based on this terminology, the calibration problem is posed as a nonlinear minimization task.

1 $${\mathbf{p}}^{*} = \arg \mathop {\min }\limits_{{\mathbf{p}}} \sqrt {\sum\limits_{j = 1}^{{N_{0} }} {\left( {y_{r0}^{(j)} - F_{CAL} \left( {y_{s0}^{(j)} ,C(y_{s0}^{(j)} ,{\mathbf{z}}_{s0}^{(j)} ,{\mathbf{p}})} \right)} \right)^{2} } }$$

The aim of (1) is to optimize the hyper-parameters of the calibration model to maximize the (*L*-square) alignment between the NO_2_ readings from the reference and corrected low-cost sensors across the training set.

### Additive and multiplicative low-cost sensor correction

**Figure 7:**
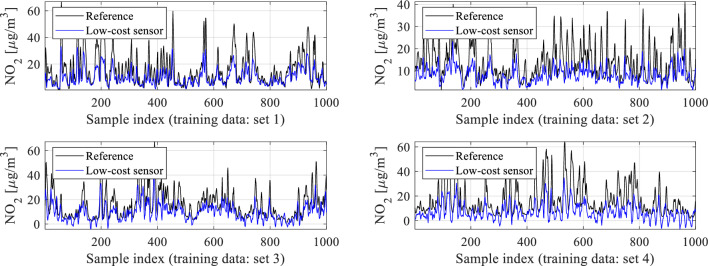
Selected reference and low-cost sensor training data subsets. A typical amplitude of low-cost sensor data variations is lower than for the reference, therefore, multiplicative scaling with coefficient *A* > 1 may be advantageous in improving the calibration process quality.

**Figure 8:**
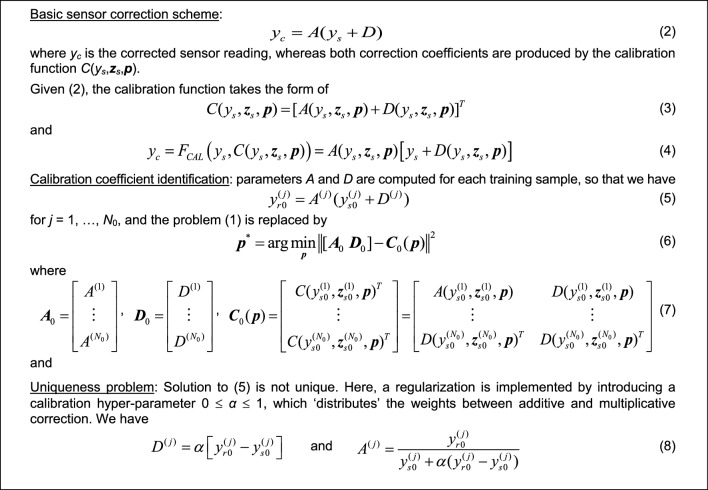
Fundamental output correction of the low-cost NO_2_ sensor: affine scaling.

Conventional correction methods often model the disparities between reference and low-cost sensor readings directly. In this study, we adopt an affine scaling approach that involves both additive and multiplicative correction. This method introduces additional degrees of freedom, enhancing the reliability of the calibration process. In our case, it is recommended to use a multiplicative scaling factor greater than one, as the typical amplitude variations in reference data are higher than those in low-cost sensor measurements, cf. Fig. 7. Details of this correction process are outlined in Fig. 8. It is essential to note that for *A*^(*j*)^ to be greater than unity, the hyper-parameter *α* must be less than unity (cf. (8)). In practice, *α* can be optimized simultaneously with training the NN calibration model (see Section "Statistical pre-processing of low-cost sensor measurements"). Through preliminary experiments, a suitable value for *α* found to be 0.8 will be utilized in our validation studies discussed in Section "Results and discussion".

As indicated in Fig. 8, the ANN model is identified based on the training data in the form of the coefficients *A* and *D* computed for each training sample. In other words, the coefficients *A*^(*j*)^ and *D*^(*j*)^ are computed for each pair of the raw sensor data *y*_*s.*0_^(*j*)^ and *y*_*r.*0_^(*j*)^ so that perfect matching is ensured as shown in (5). Subsequently, the calibration ANN model is trained to render the values of *A* and *D* for any combination of auxiliary parameters ***z***_*s*_ and primary sensor reading *y*_*s*_. The information about the reference reading at this combination is encoded in the training pairs *A*^(*j*)^, *D*^(*j*)^ combined with their corresponding sensor output *y*_*s.*0_^(*j*)^.

### Statistical pre-processing of low-cost sensor measurements

**Figure 9:**
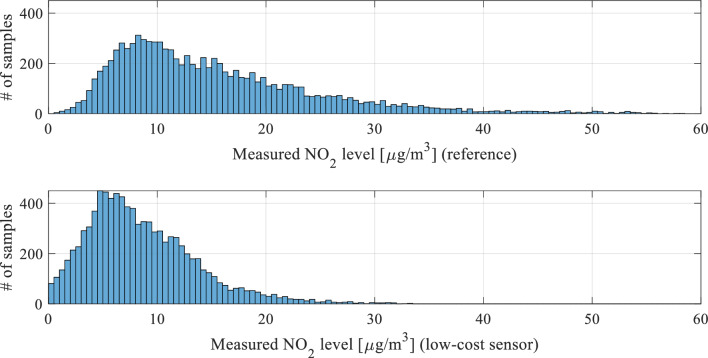
Histograms of the reference NO_2_ readings (top) and raw (uncorrected) low-cost sensor NO_2_ measurements (bottom), obtained for the complete training datasets. Note that the statistical distribution for the low-cost sensor is shifted towards lower values, which indicates that the typical readings are lower than for the reference, as also observed in Fig. 7.

One of the keystones of the proposed calibration procedure is statistical pre-processing of the low-cost sensor readings. A potential usefulness of this procedure stems from the observations made in Section "Additive and multiplicative low-cost sensor correction", specifically, the observed discrepancies between typical measured NO_2_ levels between the reference station and the low-cost sensor, as illustrated in Fig. 7. These discrepancies are well-represented on the histogram plots shown in Fig. 9. The statistical distribution of the measurements for the low-cost sensor is shifted towards lower values, which indicates that the typical readings are lower than for the reference.

The proposed pre-processing procedure aims at reducing the aforementioned misalignment by initial scaling of the low-cost sensor readings using a nonlinear transformation of the form

9 $$P(y_{s} ,{\mathbf{s}}) = P\left( {y_{s} ,[s_{1} \;s_{2} \;s_{3} ]^{T} } \right) = s_{1} + s_{2} y_{s} + s_{3} y_{s}^{2}$$

which is to be applied to all sensor measurements simultaneously. The second order polynomial has been chosen as the simplest nonlinear function that can be utilized to match the probability distributions represented by the histograms. The idea is as follows. Assuming that the probability distributions are generally similar, using affine transformation (shift + linear scaling) is generally sufficient because it allows for matching the distribution means and standard deviations. The second order has been added in order to introduce a slight nonlinearity, thereby improving the quality of histogram matching. We will also use a vector notation for *P*, i.e.,

10 $$P({\mathbf{y}},{\mathbf{s}}) = P\left( {[y_{1} \;...\;y_{N} ]^{T} ,[s_{1} \;s_{2} \;s_{3} ]^{T} } \right) = \left[ \begin{gathered} s_{1} + s_{2} y_{1} + s_{3} y_{1}^{2} \\ \vdots \\ s_{1} + s_{2} y_{N} + s_{3} y_{N}^{2} \\ \end{gathered} \right]$$

**Figure 10:**
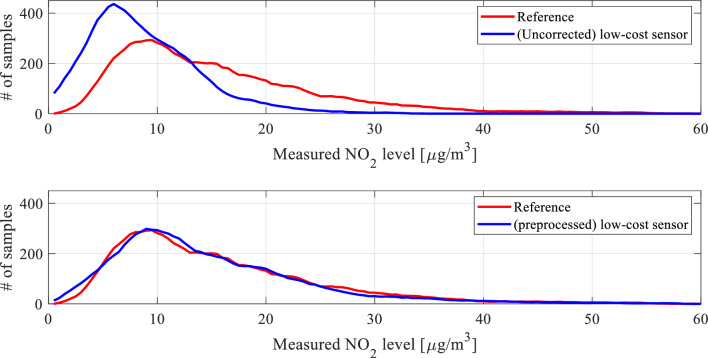
Smoothened histograms of the reference versus raw low-cost sensor (top) and the reference versus pre-processed low-cost sensor (bottom). As it can be observed, pre-processing aligns the measurement distributions of the low-cost sensor, thereby making is better prepared for further calibration.

The coefficient vector ***s*** is determined to improve the alignment of the smoothed histograms shown in Fig. 10. The latter is defined as

11 $$H({\mathbf{y}}) = \left[ {{\mathbf{z}}\;\;S({\mathbf{N}}_{{\mathbf{y}}} )} \right]$$

where

12 $${\mathbf{z}} = \left[ {z_{1} \;z_{2} \;...\;z_{M} } \right]^{T}$$

is a vector of histogram bins (i.e., intervals splitting the horizontal axis in Fig. 9 into respective compartments), whereas

13 $${\mathbf{N}}_{{\mathbf{y}}} = \left[ {n_{y.1} \;n_{y.2} \;...\;n_{y.M} } \right]^{T}$$

denotes the vector of the number of (training data) readings that fall within the respective intervals. The function *S*(⋅) represents a smoothing procedure.

Having defined the smoothed histogram, the pre-processing is accomplished by solving

14 $${\mathbf{s}}^{*} = \arg \mathop {\min }\limits_{{\mathbf{s}}} \left\| {H({\mathbf{y}}_{r} ) - H(P({\mathbf{y}}_{s} ,{\mathbf{s}}))} \right\|$$

where ***y***_*r*_ and ***y***_*s*_ stand for the aggregated reference and low-cost sensor NO_2_ readings.

Note that if the histogram bins ***z*** are identical for the reference and the sensor (which is assumed here), the functional in (14) boils down to comparing the respective *S*(***N***_*y*_) vectors. Solving problem (14) is equivalent to matching the smoothed histograms of the reference and pre-processed low-cost sensor histograms. The unknown variables in this process are the scaling polynomial coefficients, that is, the vector ***s*** defined in Eq. (9). Note that the matching is not performed for the number of observations falling into the reference bins as these are discrete numbers, and solving least-square regression problem would be problematic when using gradient-based routines. Instead, matching is performed upon smoothed histograms, which are continuous functions of the bin indices. The process (14) is effectively fitting the second-order polynomial that determines the histogram scaling.

**Figure 11:**
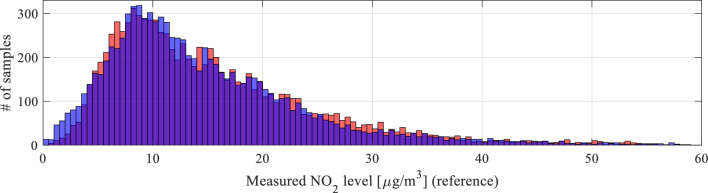
A comparison between the reference data (red) and pre-processed (blue) low-cost sensor histogram. Good alignment between the two datasets can be observed. Overlapping data marked purple.

**Figure 12:**
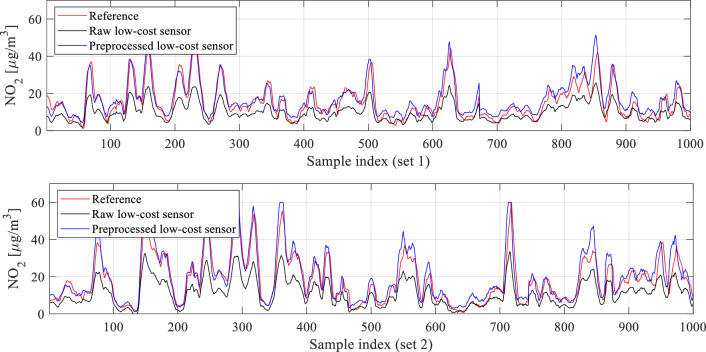
The effects of statistical pre-processing illustrated for two selected subsets of the training data. As it can be observed, pre-processing leads to a significant improvement of correlation between the reference and low-cost sensor readings.

Figure 10 shows the smoothed histograms before (top) and after pre-processing (bottom), indicating considerable improvement in terms of the alignment. Direct comparison between raw (non-smoothed) histograms can be found in Fig. 11. Figure 12 shows the effects of pre-processing for selected subsets of the training data. As mentioned earlier, pre-processing will be employed as the first calibration step, followed by surrogate-predicted correction to be discussed from Section "Sensor calibration using neural network surrogate" on.

### Sensor calibration using neural network surrogate

**Figure 13:**
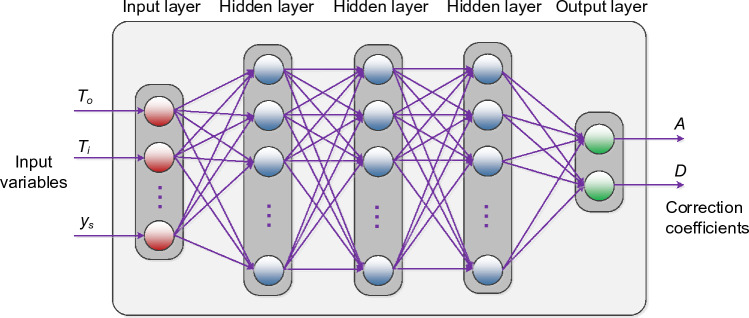
ANN surrogate used as the core calibration model. Here, we employ a multi-layer perceptron (MLP) with three fully-connected hidden layers. When statistical data pre-processing it utilized (cf. Section "Statistical pre-processing of low-cost sensor measurements"), then the input *y*_*s*_ of the primary sensor reading is not taken directly from the sensor. Instead, it is a pre-processed value.

The primary calibration model employed in this study is an artificial neural network (ANN) surrogate. Specifically, we have opted for a multi-layer perceptron (MLP) architecture^51,52^ featuring three fully connected hidden layers, each consisting of twenty neurons utilizing a sigmoid activation function, as illustrated in Fig. 13. The model's hyper-parameters are identified using a backpropagation Levenberg–Marquardt algorithm^53^ (setup: 1000 learning epochs, performance evaluation using mean-square error (MSE), randomized training/testing data division). It should be emphasized that the aforementioned data division is pertinent to the training data itself (i.e., the training data is internally split into ‘training’ and ‘validation’ data for the purpose of ANN training in each epoch). The testing data as specified in Fig. 5 is kept separate and only used for model validation in the numerical experiments in Section "Results and discussion".

We deliberately chose a relatively simple ANN architecture to expedite the training process and prioritize its role as a regression model. Given the ample training samples available, the model's sensitivity to the number of layers and neurons is limited. Furthermore, this streamlined architecture effectively mitigates inherent noise present in both the reference and sensor readings.

The calibration model takes inputs comprising environmental factors (internal/external temperature, humidity, etc.) and NO_2_ measurements from both the primary and auxiliary sensors. The outputs of the neural network (NN) model are the affine scaling coefficients *A* and *D*. In Section "Calibration model inputs", we delve into diverse extended input sets aimed at bolstering the calibration process's reliability. The effects of these expanded sets, alongside the consequences of restricting inputs to various subsets of the vector ***z***_*s*_, will be analysed in Section "Results and discussion" to assess how input configuration impacts the efficacy of calibration.

### Calibration model inputs

In this section, we discuss various input configurations of the ANN calibration model. Section "Calibration input configuration I: basic setup" recalls the basic parameter set discussed earlier. The extended input set, integrating differentials of environmental variables and primary NO_2_ readings, is explored in Section "Calibration input configuration II: differentials".

Section "Calibration input configuration III: time series of prior NO2 measurements" analyses the final setup that involves time series of prior NO_2_ measurements from the low-cost sensor. In our investigations, we focus on potential benefits of particular setups in terms of improving the calibration process dependability.

#### Calibration input configuration I: basic setup

The fundamental configuration of the calibration model inputs includes the auxiliary data vector ***z***_*s*_ = [*T*_*o*_* T*_*i*_* H*_*o*_* H*_*i*_* P S*_1_ *S*_2_]^*T*^. This set of values comprises external/internal temperature, humidity, atmospheric pressure, and NO_2_ data from redundant sensors. These elements are augmented by the primary sensor's NO_2_ measurements, *y*_*s*_. Section "Results and discussion" will further investigate constrained variations of this arrangement to determine the individual elements' significance.

#### Calibration input configuration II: differentials

The basic input arrangement elucidated in Section "Calibration input configuration I: basic setup" can be extended by incorporating additional parameters representing local (temporal) fluctuations in environmental variables and NO_2_ readings. More specifically, we define differentials

15 $$\Delta y_{s}^{(j)} = \frac{{y_{s}^{(j)} - y_{s}^{(j)} ( - \Delta t)}}{\Delta t}$$

where Δ*t* is the time interval between subsequent sensor readings; *y*_*s*_^(*j*)^(–Δ*t*) stands for the last measurement taken before *y*_*s*_^(*j*)^. Differentials of the environmental parameters are defined in a similar manner

16 $$\Delta T_{o}^{(j)} = \frac{{T_{o}^{(j)} - T_{o}^{(j)} ( - \Delta t)}}{\Delta t},\,\,\,\Delta T_{i}^{(j)} = \frac{{T_{i}^{(j)} - T_{i}^{(j)} ( - \Delta t)}}{\Delta t}$$

17 $$\Delta H_{o}^{(j)} = \frac{{H_{o}^{(j)} - H_{o}^{(j)} ( - \Delta t)}}{\Delta t},\,\,\Delta H_{i}^{(j)} = \frac{{H_{i}^{(j)} - H_{i}^{(j)} ( - \Delta t)}}{\Delta t}$$

18 $$\Delta P_{{}}^{(j)} = \frac{{P_{{}}^{(j)} - P_{{}}^{(j)} ( - \Delta t)}}{\Delta t}$$

Note that computing (15), (16), (17), (18) only requires storing one extra set of readings. The differentials, especially Δ*y*_*s*_^(*j*)^, quantify local fluctuations in NO_2_ level, which facilitates prediction of forthcoming alterations. Moreover, integrating differentials of environmental variables can provide explicit or implicit insights into the dynamics of relevant factors such as cross-sensitivity to other gases. This addition of differentials as supplementary inputs into the NN surrogate allows exploration of their potential contribution to enhancing the calibration quality.

**Figure 14:**
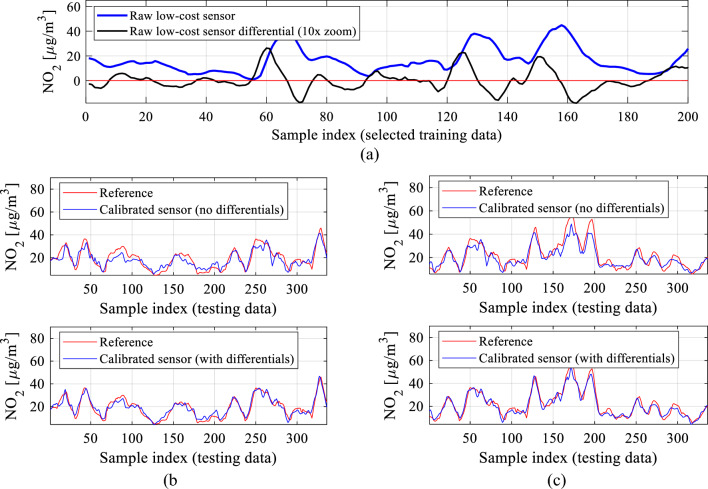
Differentials used as additional ANN surrogate inputs to enhance calibration dependability: (**a**) selected training data sequence (NO_2_ readings from the low-cost sensor) and its corresponding differentials (15); (**b**) the effects of incorporating differentials shown for a selected sequence of testing data; (**c**) the effects of differentials shown for another testing data sequence. Note that including differentials (here, of all environmental variables and the primary NO_2_ readings from the low-cost sensor) noticeably improves data alignment.

**Figure 15:**
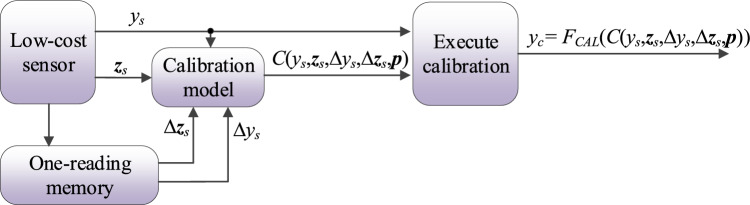
Calibration of the low-cost sensor with differentials used as additional calibration model inputs. Auxiliary data and sensor output *y*_*s*_ are used to obtain the correction coefficients *C*(*y*_*s*_,***z***_*s*_,Δ*y*_*s*_,Δ***z***_*s*_,***p***), used to compute the corrected sensor output *y*_*c*_. The pre-processing step is not shown for clarity.

A visual illustration has been provided in Fig. 14. In particular, Fig. 14a shows—for a selected sequence of the training data—the NO_2_ readings from the low-cost sensor alongside the respective differentials. Meanwhile, Fig. 14b and c, demonstrate the effects of incorporating the differentials as auxiliary calibration model inputs. The flow diagram of the modified calibration process involving differentials can be found in Fig. 15.

#### Calibration input configuration III: time series of prior NO_2_ measurements

Expanding the concept of differentials might involve integrating an extended series of previous sensor measurements, which may not be suitable for mobile monitoring platforms but could significantly enhance the calibration of stationary systems, like the one discussed in Section "Autonomous NO2 monitoring platform". The additional inputs for the calibration surrogate comprise

19 $$y_{s}^{(j)} ( - s\Delta t),\,\,\,s\, = \,1,\,2,\, \ldots ,\,N_{s} .$$

In (19), Δ*t* is the reading time interval, whereas *N*_*s*_ is the number of prior measurements used as extra inputs. Although a natural choice for incorporating a time series such as (19) would be recurrent neural networks (RNN)^54^, in our case, *N*_*s*_ will be fixed throughout making feedforward networks a sufficient representation. Note that *N*_*s*_ = 1 is equivalent to the incorporation of differentials described in Section "Calibration input configuration II: differentials".

**Figure 16:**
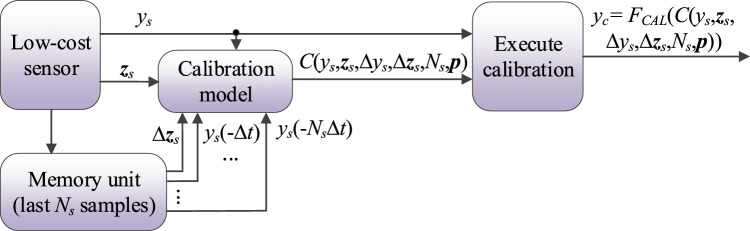
Calibration of the low-cost sensor with time series of prior measurements used as additional calibration model inputs. Auxiliary data are used to obtain the correction coefficients *C*(*y*_*s*_,***z***_*s*_,Δ*y*_*s*_,Δ***z***_*s*_,*N*_*s*_,***p***), used to compute the corrected sensor output *y*_*c*_. The pre-processing step is not shown for clarity.

**Figure 17:**
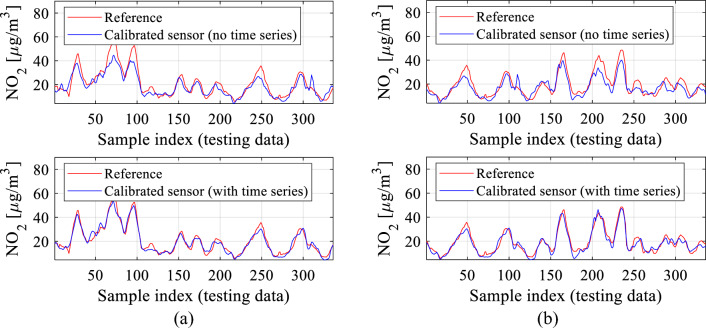
The effects of incorporating a time series of length *N*_*s*_ = 3 of prior NO_2_ readings into the NN calibration model, along with the environmental parameters differentials. Shown are reference and calibrated low-sensor data without and with the mentioned time series, obtained for two selected sequences of the testing data: (**a**) first sequence, (**b**) second sequence.

The extended flow diagram of the calibration procedure involving the time series of length *N*_*s*_ has been shown in Fig. 16. Figure 17 demonstrates the advantages of including short time series as auxiliary calibration model inputs for *N*_*s*_ = 3. Section "Results and discussion" will carry out a comprehensive analysis of the effects of the length *N*_*s*_ on calibration process reliability.

### Global data scaling

**Figure 18:**
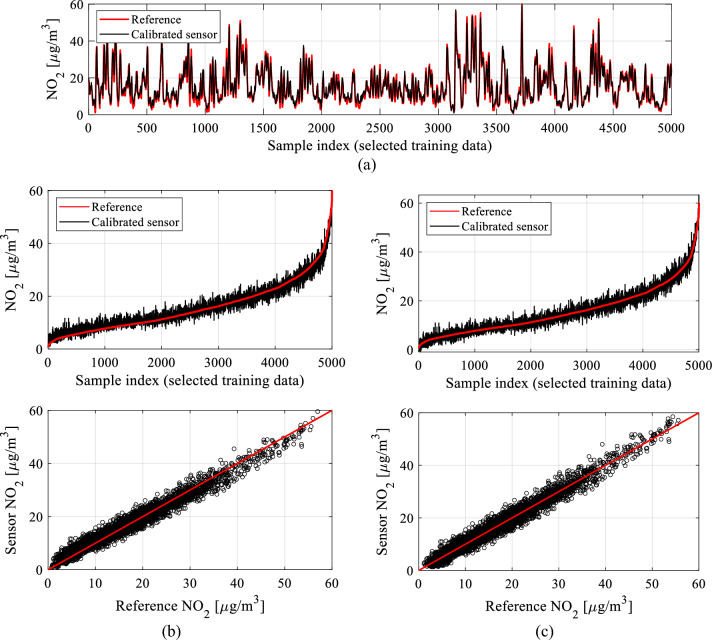
Global response correction: (**a**) a subset of selected training data; (**b**) the same data arranged based on increasing NO_2_ reference readings (top) accompanied by the corresponding scatter plot (bottom). Despite the apparent alignment showcased in Fig. 18a, there is an observable systematic offset dependent on the level; (**c**) the same data after the application of global data scaling, showcasing a notable decrease in the systematic offset and an enhancement in the symmetry of the scatter plot. In this instance, global correction results in an improved correlation coefficient, rising from 0.93 to 0.95, and a reduction in RMSE from 2.1 to 1.8 µg/m^3^.

The last algorithmic component integrated into the proposed calibration process involves global data scaling. This approach adjusts the correction coefficients anticipated by the ANN surrogate based on the current values of environmental factors, NO_2_ measurements from both primary and redundant sensors, potential differentials, and a time series of *N*_*s*_-length primary NO_2_ data. The surrogate aims to minimize the disparity between the reference and low-cost sensor data in the least-square sense (cf. (1)). Yet, resolving (1) might reveal certain systematic discrepancies reliant on the measured NO_2_ level, as depicted in Fig. 18a and b for a specific subset of training data. This distinction becomes apparent when examining the data sorted by reference NO_2_ levels and through the scatter plot's slight skew seen in the bottom panel of Fig. 18b.

**Figure 19:**
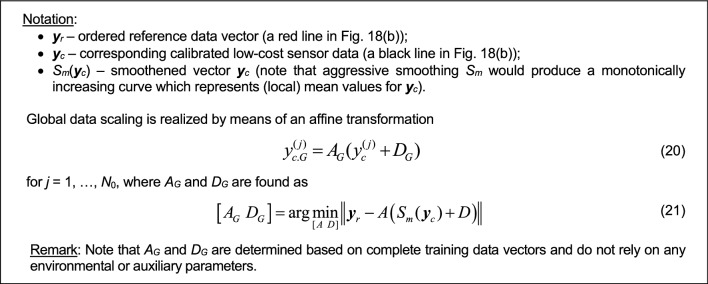
Global response correction through affine transformation of the ordered NO_2_ data from the calibrated low-cost sensor.

The global data scaling aims at reducing the discussed offsets by means of an affine transformation of the smoothed sensor measurements. In plain words, it corresponds to a ‘rotation’ of the scatter plot rendering it less skewed with respect to the identify mapping. A rigorous formulation of the process has been explained in Fig. 19. Coefficients *A*_*G*_ and *D*_*G*_ are determined from the complete dataset; they are not functions of the environmental or auxiliary parameters.

The impact of implementing global data scaling is evident in Fig. 18c. In the depicted case, there is a noticeable reduction in the offset and an enhanced symmetry within the scatter plot. Simultaneously, the correlation coefficient improves from 0.93 to 0.95, while the RMSE decreases from 2.1 to 1.8 µg/m^3^ based on the training data. Although its advantages might be somewhat constrained for the testing data, global data scaling still proves beneficial, as shown in Section "Results and discussion".

Again, it should be noted that that the global data correction is a separate stage, which is applied after calibrating the sensor using the scaling coefficients *A* and *D* rendered by the ANN model. The inputs of the ANN model are the auxiliary parameters (vector ***z***_*s*_), the primary sensor measurement *y*_*s*_, and (optionally) the differentials and the time series of prior measurements.

The ANN model produces coefficients *A* and *D* being functions of these input variables and applies them to the low-cost sensor readings as in (2). The global correction (20) is applied afterwards using coefficients *A*_*G*_ and *D*_*G*_ obtained for the entire training dataset (i.e., not being functions of individual measurements). These coefficients are the same for all samples underdoing the global correction process.

### Operating flow of NO_2_ monitoring by means of calibrated sensor

**Figure 20:**
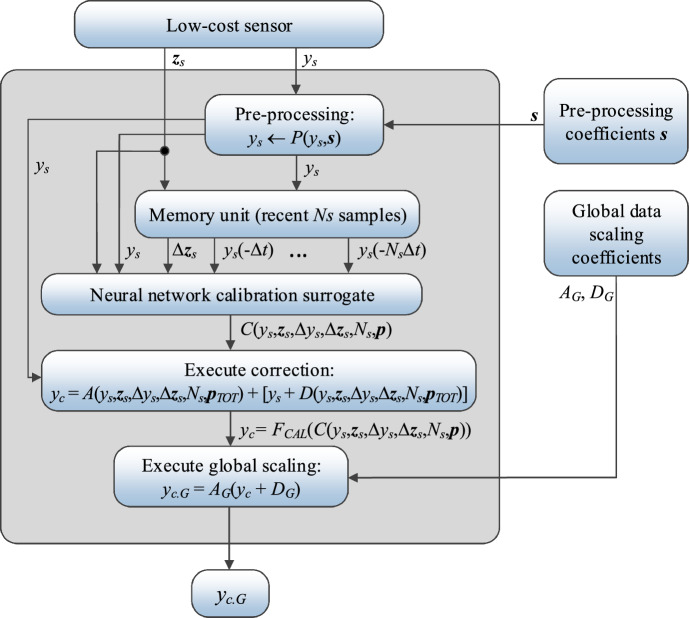
Low-cost sensor calibration procedure as proposed in this study. Pre-processing of the sensor readings is followed by generating (local) calibration coefficients using the ANN surrogate (based on the auxiliary vector ***z***_*s*_, the actual NO_2_ reading *y*_*s*_ from the low-cost sensor, their differentials, as well as a short-term time series of prior nitrogen dioxide readings from the primary sensor). The affine scaling is then applied to the sensor reading to produce the outcome *y*_*c*_. Subsequently, global response correction is superimposed to produce the final corrected reading *y*_*c.G*_.

Below, we summarize the operation of the complete calibration process of the low-cost sensor. The procedure combines the correction mechanisms detailed in Sections "Additive and multiplicative low-cost sensor correction" through "Global data scaling". The first step is pre-processing elucidated in Section "Statistical pre-processing of low-cost sensor measurements", where the overall distributions of the sensor and the reference data are aligned. Subsequently, the ANN surrogate predicts the (local) correction coefficients using the auxiliary vector ***z***_*s*_ and NO_2_ reading *y*_*s*_ from the low-cost sensor, their differentials, as well as an *N*_*s*_-long time series of prior NO_2_ measurements from the primary sensor. The intermediate outcome *y*_*c*_ is obtained by applying the affine correction (2), (3). The last stage is global data scaling (20), (21), which produces the final corrected NO_2_ reading. A flow diagram of the process has been shown in Fig. 20.

## Results and discussion

This section concentrates on validating the proposed calibration method for the low-cost sensor, applied to the autonomous monitoring platform detailed in Section "Autonomous NO2 monitoring platform". The content is organized as follows. Section "Reference and low-cost sensor datasets" discusses the reference and low-cost sensor datasets. Section "Results" presents results obtained from various calibration setups explored in comparative experiments. Finally, Section "Discussion" summarizes findings and discusses the performance of the calibration process.

### Reference and low-cost sensor datasets

**Figure 21:**
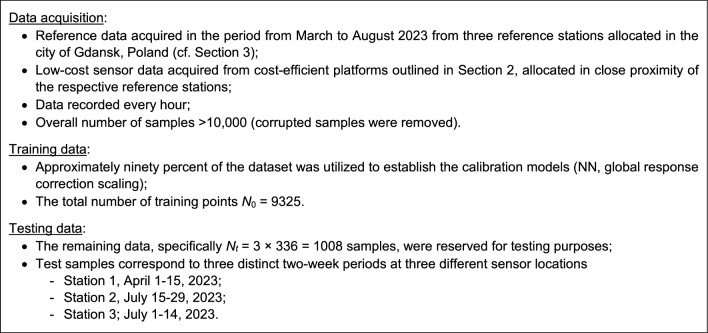
Characterization of the training and testing data acquired to carry out calibration of the low-cost sensor of Section "Autonomous NO2 monitoring platform".

**Figure 22:**
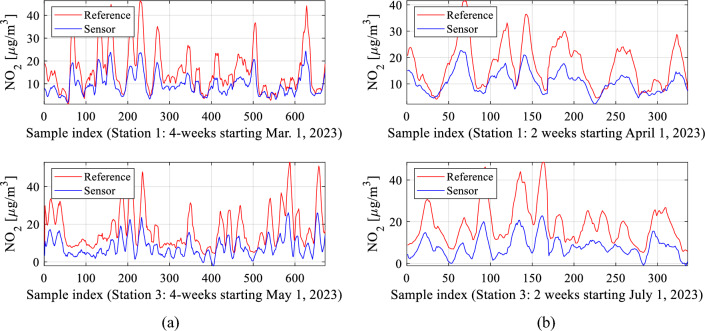
Selected subsets of NO_2_ readings from the reference stations and the raw (uncorrected) low-cost sensors: (**a**) training data, (**b**) testing data.

The proposed calibration procedure has been validated using the datasets acquired from the reference stations (as outlined in Section "Reference data. Public monitoring stations") and the monitoring platforms (detailed in Section "Autonomous NO2 monitoring platform"). The data was collected hourly between March and August 2023, cf. Figure 21. For the sake of illustration, Fig. 22 presents selected subsets of the reference and uncorrected low-cost sensor training and testing data. Significant disparities between the readings from the reference and the sensor can be observed, which poses a considerable challenge for the calibration process.

## Results

In this analysis, we delve into the calibration outcomes of the low-cost NO_2_ sensor within the monitoring platform highlighted in Section "Autonomous NO2 monitoring platform". We explore various setups of the calibration model inputs to assess the importance of specific algorithmic elements within the correction scheme. Additionally, we selectively enable or disable auxiliary mechanisms, i.e., pre-processing and global data scaling for some configurations. Table 1 presents all the scrutinized setups. Each configuration undergoes ten independent training cycles, and the model with the optimal set of hyper-parameters is chosen as the final model.

**Table 1:** Input setups of the calibration model considered in verification experiments.

Calibration setup	Calibration input variables					Global response correction	Pre-processing
	Auxiliary data	NO_2_ reading from primary sensor (*y*_*s*_)	Differentials		Time series *y*_*s*_(–Δ*t*),…,* y*_*s*_(–*N*_*s*_Δ*t*)		
			Δ***z***_*s*_	Δ*y*_*s*_			
A.1	Restricted (*T*_*o*_, *T*_*i*_, *H*_*o*_, and *H*_*i*_)	×	×	×	×	×	×
A.2	Restricted (***z***_s_ without *P*)	×	×	×	×	×	×
A.3	Restricted (***z***_s_ without *P*)	✓	×	×	×	×	×
A.4	Complete ***z***_*s*_	✓	×	×	×	×	×
A.5	Complete ***z***_*s*_	✓	×	×	×	✓	×
A.6	Complete ***z***_*s*_	✓	×	✓	×	✓	×
A.7	Complete ***z***_*s*_	✓	✓	✓	×	×	×
A.8	Complete ***z***_*s*_	✓	✓	✓	×	✓	×
B.1	Complete ***z***_*s*_	✓	✓	×	✓ (*N*_*s*_ = 0)	×	×
B.2	Complete ***z***_*s*_	✓	✓	×	✓ (*N*_*s*_ = 2)	×	×
B.3	Complete ***z***_*s*_	✓	✓	×	✓ (*N*_*s*_ = 4)	×	×
B.4	Complete ***z***_*s*_	✓	✓	×	✓ (*N*_*s*_ = 6)	×	×
B.5	Complete ***z***_*s*_	✓	✓	×	✓ (*N*_*s*_ = 8)	×	×
C.1	Complete ***z***_*s*_	✓	✓	×	✓ (*N*_*s*_ = 0)	✓	×
C.2	Complete ***z***_*s*_	✓	✓	×	✓ (*N*_*s*_ = 2)	✓	×
C.3	Complete ***z***_*s*_	✓	✓	×	✓ (*N*_*s*_ = 4)	✓	×
C.4	Complete ***z***_*s*_	✓	✓	×	✓ (*N*_*s*_ = 6)	✓	×
C.9	Complete ***z***_*s*_	✓	✓	×	✓ (*N*_*s*_ = 8)	✓	×
D.1	Complete ***z***_*s*_	✓	✓	×	✓ (*N*_*s*_ = 0)	✓	✓
D.2	Complete ***z***_*s*_	✓	✓	×	✓ (*N*_*s*_ = 2)	✓	✓
D.3	Complete ***z***_*s*_	✓	✓	×	✓ (*N*_*s*_ = 4)	✓	✓
D.4	Complete ***z***_*s*_	✓	✓	×	✓ (*N*_*s*_ = 6)	✓	✓
D.5	Complete ***z***_*s*_	✓	✓	×	✓ (*N*_*s*_ = 8)	✓	✓

The calibration setups under examination are divided into four groups, denoted as A to D. The first group encompasses configurations that do not utilize the time series of previous NO_2_ measurements. The second group involves setups that incorporate time series of past readings, varying in length (*N*_*s*_), excluding global response correction. The third group combines time-series-based calibration with global data scaling. The final group incorporates pre-processing as detailed in Section "Statistical pre-processing of low-cost sensor measurements". Experimenting with different *N*_*s*_ values enables us to identify the most effective time series length.

**Figure 23:**
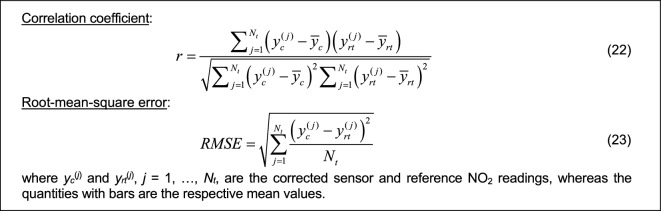
Definitions of the correlation coefficient *r* and RMSE.

**Figure 24:**
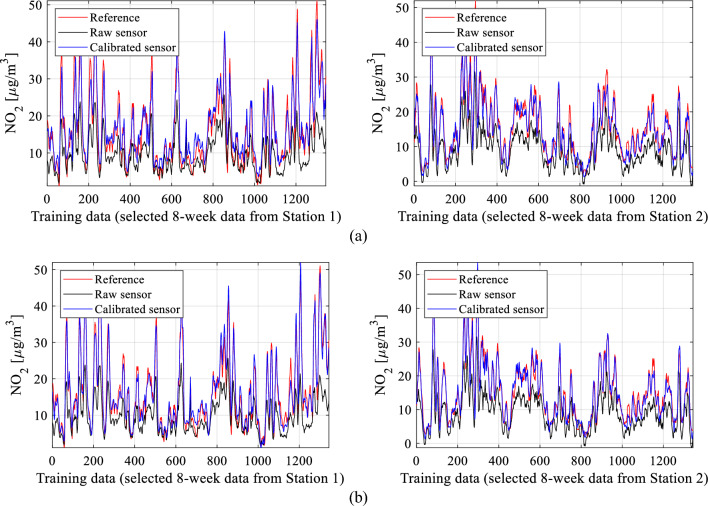
Sensor calibration performance for selected subsets of the training data: (**a**) setup B.4, (**b**) setup D.3.

**Figure 25:**
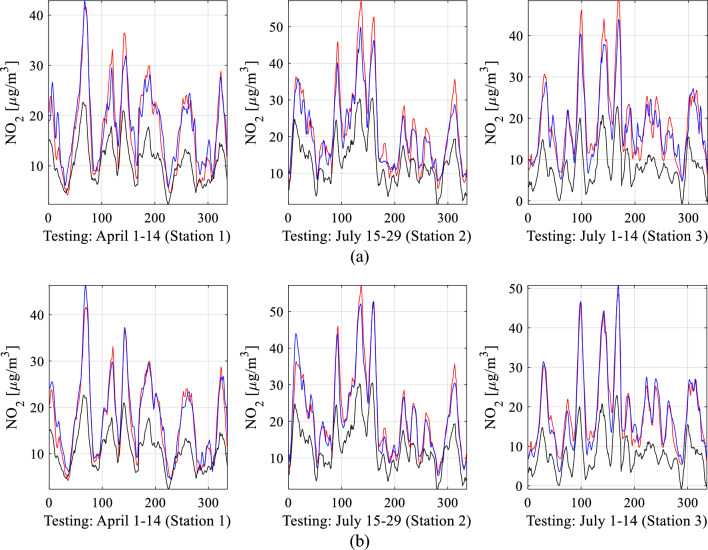
Sensor calibration performance for selected subsets of the testing data: (**a**) setup B.4, (**b**) setup D.3.

**Figure 26:**
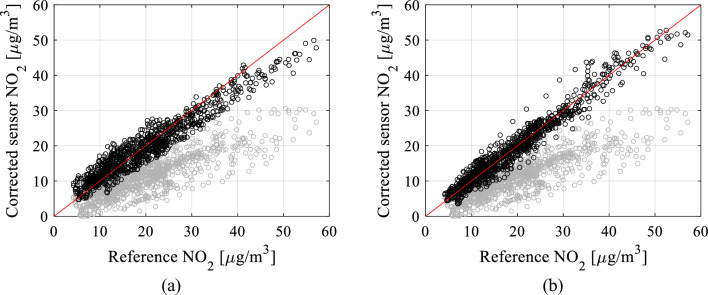
Scatter plots for the testing data (uncorrected—gray, corrected—black): (**a**) setup B.4, (**b**) setup D.3.

**Figure 27:**
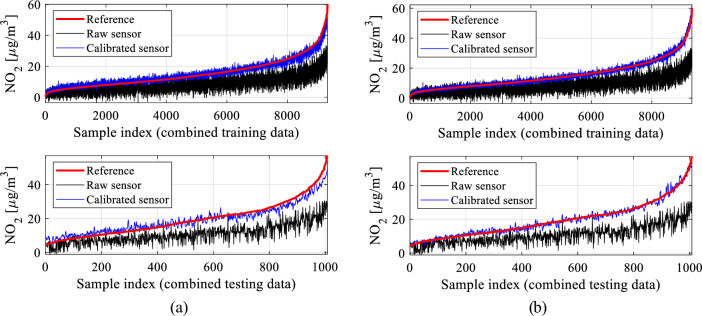
Performance of sensor calibration for: (**a**) setup B.4, (**b**) setup D.3. Shown are the entire training dataset (top) and testing dataset (bottom), arranged in ascending order according to NO_2_ reference readings. Note substantial enhancement achieved through calibration, i.e., bringing the calibrated sensor readings much closer to their corresponding reference measurements compared to the raw data.

The results from all calibration setups are consolidated in Table 2, encompassing the correlation coefficient and modeling error (RMSE) for both training and testing data (see Fig. 23 for definitions). To streamline the presentation, data visualization is provided for four specific calibration setups: B.4, and D.3. Figure 24 displays the reference, raw low-cost sensor, and calibrated sensor NO_2_ measurements (training data) for two chosen eight-week periods. Figure 25 illustrates the same information for testing data across three two-week periods, while Fig. 26 showcases scatter plots for the testing data. Finally, Fig. 27 presents NO_2_ measurements for setups B.4, and D.3 based on ascending reference readings.

**Table 2:** Sensor calibration performance: correlation coefficients and RMSE.

Calibration setup	Training data		Testing data	
	Correlation oefficient *r*	RMSE [μg/m^3^]	Correlation coefficient *r*	RMSE [μg/m^3^]
A.1	0.82	4.0	0.70	5.6
A.2	0.89	3.0	0.81	4.3
A.3	0.91	2.8	0.84	4.0
A.4	0.93	2.5	0.86	3.9
A.5	0.94	2.4	0.878	3.6
A.6	0.93	2.6	0.886	3.4
A.7	0.93	2.4	0.896	3.3
A.8	0.94	2.3	0.903	3.17
B.1	0.92	2.6	0.892	3.3
B.2	0.93	2.4	0.907	3.1
B.3	0.93	2.4	0.907	3.1
B.4	0.93	2.5	0.903	3.2
B.5	0.93	2.5	0.900	3.3
C.1	0.92	2.5	0.905	3.1
C.2	0.93	2.4	0.921	2.9
C.3	0.93	2.4	0.923	2.8
C.4	0.93	2.4	0.917	2.9
C.5	0.93	2.5	0.912	3.1
D.1	0.94	2.0	0.932	2.6
D.2	0.95	1.9	0.944	2.4
D.3	0.96	1.8	0.947	2.4
D.4	0.96	1.8	0.947	2.4
D.5	0.96	1.8	0.945	2.4

## Discussion

The experiments in Section "Results" aimed to verify the effectiveness of the proposed calibration process. One crucial aspect under examination was whether the correction strategy introduced could adequately align the reference and low-cost sensor readings, ensuring reliable monitoring of nitrogen dioxide. Furthermore, we aimed at verifying the relevance of correction mechanisms, specifically, the pre-processing and global data scaling procedures, and benefits of incorporating environmental parameter differentials, and time series of prior NO_2_ readings from the low-cost sensor as additional calibration inputs. We were also interested in identifying the optimal length *N*_*s*_ of this series. It is also important to recall that the initial discrepancies between the low-cost sensor and the reference measurements are significant, whereas the NO_2_ level changes considerably (from almost zero to sixty µg/m^3^) and often quickly, which make the calibration a challenging endeavour.

The findings in Table 2 showcase the exceptional performance of the proposed calibration technique. Among the calibration setups assessed, the most effective configurations belong to group D, specifically D.3 and D.4. These setups integrate all correction mechanisms outlined in Section "Precise sensor calibration using statistical pre-processing, ANN surrogates, and global data scaling", encompassing pre-processing, global data scaling, and leveraging extended input variables covering environmental parameters, auxiliary NO_2_ readings, differentials, and medium-length time series (*N*_*s*_ ranging between four and six). For instance, in setup D.3, the correlation coefficient reaches approximately 0.95, with an RMSE of 2.4 µg/m^3^ for the testing data. Moreover, the average relative RMS error is merely around 11 percent. The precision of the calibrated sensor is evident in its excellent alignment with the reference data, as observed in both the training (Fig. 24d) and testing data (Fig. 25d). The reported numbers are particularly impressive when compared to the metrics of the raw (uncorrected) sensor, which are as follows: correlation coefficients 0.07 and 0.04 (training and testing data, respectively), and RMSE of 8.9 and 10.8 µg/m^3^ (training and testing data, respectively).

A review of the results across various calibration setups underscores the significance of each incorporated correction mechanism. For instance, augmenting the inputs in the calibration model significantly impacts both the correlation coefficient and RMSE. Comparing configurations A.1, A.2, A.3, A.4, and A.7 (excluding global response correction) highlights this, where the correlation coefficient improves from 0.7 to 0.89, and RMSE drops from 5.6 to 3.4 µg/m^3^. Consistent integration of global response correction consistently bolsters the correlation coefficient by nearly 0.02 and reduces RMSE by about 0.2 µg/m^3^ (e.g., comparing setup A.5 versus A.4, or C.1 versus B.1).

Introducing time series data further enhances results, achieving up to a 0.03 improvement in correlation coefficient and a reduction of 0.3 µg/m^3^ in RMSE (e.g., setups C.3 or C.4). Moreover, data pre-processing significantly contributes to calibration enhancements by adding up to 0.03 to the correlation coefficient and reducing RMSE by nearly 0.3 µg/m^3^. These improvements are visually evident in Figs. 24, 25, and 26, where transitioning from simpler configurations to more advanced ones (e.g., B.4 and D.3) noticeably improves alignment between the reference and corrected low-cost sensor readings. Additionally, it centres the scatter plots closer to the identity function.

The enhancements in reliability are also visually highlighted in Fig. 27, where both training and testing data are arranged by ascending reference NO_2_ levels. Moving from the simpler setup A.2 through intermediate stages (A.7 and B.4) to the advanced configuration D.3 significantly reduces deviations between the reference and calibrated sensor readings. An in-depth analysis of setups B and C reveals that the most favourable configuration in terms of the time series length is *N*_*s*_ = 4, showcasing the highest correlation coefficient and minimal RMSE. However, with the inclusion of pre-processing (setups D), the impact of *N*_*s*_ becomes less distinctive, suggesting that the calibration performance becomes more resilient to variations in this parameter.

Additional experiments were conducted to verify the effects of including auxiliary NO_2_ sensor readings as supplementary calibration inputs. The considered setups are listed in Table 3. The results are encapsulated in Table 4. Note that setups E.1 and E.5 were previously considered as Cases E.1 and E.3 in Table 1. These are repeated to ensure completeness of the data in Tables 3 and 4. Note that incorporating auxiliary NO_2_ sensor data does improve the calibration process dependability. Also, it can be observed that the second auxiliary sensor *S*_2_ has a slightly higher impact, as it can be inferred from the values of correlation coefficient and RMSE. On the other hand, when the auxiliary sensors are not utilized, data alignment degrades noticeably (cf. setup E.2 versus E.3, E.4, or E.5). Furthermore, including the primary sensor measurements is also important.

**Table 3:** Verification case studies: calibration model setup.

Case	Calibration model	Calibration input variables
E.1	ANN (= Case A.1 of Table 2)	Restricted auxiliary data (*T*_*o*_, *T*_*i*_, *H*_*o*_, and *H*_*i*_)
E.2	ANN	Restricted auxiliary data (*T*_*o*_, *T*_*i*_, *H*_*o*_, and *H*_*i*_) and *y*_*s*_
E.3	ANN	Restricted auxiliary data (*T*_*o*_, *T*_*i*_, *H*_*o*_, and *H*_*i*_), *S*_1_ and *y*_*s*_
E.4	ANN	Restricted auxiliary data (*T*_*o*_, *T*_*i*_, *H*_*o*_, and *H*_*i*_), *S*_2_ and *y*_*s*_
E.5	ANN (= Case A.3 of Table 2)	Restricted auxiliary data (*T*_*o*_, *T*_*i*_, *H*_*o*_, and *H*_*i*_), *S*_1_, *S*_2_ and *y*_*s*_

**Table 4:** Sensor calibration performance for calibration scenarios listed in Table 3.

Case	Training data		Testing data	
	Correlation coefficient *r*^2^	RMSE [μg/m^3^]	Correlation coefficient *r*^2^	RMSE [μg/m^3^]
E.1	0.82	4.0	0.70	5.6
E.2	0.88	3.2	0.81	4.4
E.3	0.90	3.0	0.84	4.1
E.4	0.89	3.0	0.85	4.0
E.5	0.91	2.8	0.84	4.0

For supplementary validation, the calibration approach introduced in this paper has been compared to several benchmark methods, specifically, linear regression, neural-network-based calibration, as well as calibration implemented using a convolutional neural network (CNN)^55^. In the case of ANN/CNN, the neural network predicts the calibrated model output directly instead of rendering the correction coefficients. Linear regression is a model of the form

22 $$S({\mathbf{z}}_{s} ) = \alpha_{0} + \alpha_{1} T_{o} + \alpha_{2} T_{i} + \alpha_{3} H_{o} + \alpha_{4} H_{i} + \alpha_{5} S_{1} + \alpha_{6} S_{2}$$

when using vector ***z***_*s*_ as calibration input, and

23 $$S_{y} ({\mathbf{z}}_{s} ,y_{s} ) = \alpha_{0} + \alpha_{1} T_{o} + \alpha_{2} T_{i} + \alpha_{3} H_{o} + \alpha_{4} H_{i} + \alpha_{5} S_{1} + \alpha_{6} S_{2} + \alpha_{7} y_{s}$$

when using extended calibration inputs (i.e., primary sensor data). The coefficients in (22) and (23) are found through least-square regression based on the training data. The ANN uses the same architecture as described in Section "Precise sensor calibration using statistical pre-processing, ANN surrogates, and global data scaling". CNN architecture is uses filters of the size 4 × 1 × 1, three convolution layers of spatial sizes 32, 16, and 8, followed by a fully connected layer of the size 64 neurons (version I), layers of sizes 64, 32, 16 (version II), and 126, 64, and 32 (version III), as well as batch normalization and ReLU layers in between the convolution layers. CNN is trained using the ADAM’s algorithm with a mini batch size of 1000 [70]. Table 5 gathers the numerical results. It should be noted that the calibration methodology proposed in this study provides significantly better results, both in terms of correlation coefficients and RMSE. Utilization of affine correction (cf. Table 2) is superior to direct prediction of the calibrated sensor when using ANN of the same architecture as well as CNN.

**Table 5:** Comparative studies: linear regression and direct ANN/CNN-based prediction.

Calibration method	Training data		Testing data	
	Correlation coefficient *r*^2^	RMSE [μg/m^3^]	Correlation coefficient *r*^2^	RMSE [μg/m^3^]
Linear regression *S*(***z***_*s*_)	0.28	7.8	0.07	9.9
Linear regression *S*_*y*_(***z***_*s*_,*y*_*s*_)	0.66	5.4	0.56	6.8
Direct ANN-based prediction (***z***_*s*_)	0.77	4.4	0.26	8.8
Direct ANN-based prediction (***z***_*s*_ and *y*_*s*_)	0.83	3.8	0.61	6.4
Direct CNN-based prediction (***z***_*s*_ and *y*_*s*_) (convolution layers: 32, 16, 8)	0.50	6.5	0.29	8.6
Direct CNN-based prediction (***z***_*s*_ and *y*_*s*_) (convolution layers: 64, 32, 16)	0.72	4.8	0.45	7.6
Direct CNN-based prediction (***z***_*s*_ and *y*_*s*_) (convolution layers: 128, 64, 32)	0.77	4.5	0.42	7.7

In summary, the showcased calibration approach proves remarkably effective. The corrected low-cost sensor measurements closely align with the reference readings, particularly in the advanced configurations, such as D.3, representing the optimal calibration setup. In practical terms, this sensor correction can be integrated offline or implemented within the platform using its on-board computational resources, as outlined in Section "Autonomous NO2 monitoring platform".

## Conclusion

This article introduced an innovative methodology for high-efficiency calibration of affordable nitrogen dioxide sensors. The proposed technique integrates various correction mechanisms, encompassing data pre-processing, additive and multiplicative response adjustments executed by an artificial neural network (ANN) surrogate, and global data scaling. The pre-processing step focuses on aligning the distribution of low-cost sensor readings across the entire training dataset with reference measurements. Utilizing the ANN surrogate, the method predicts specific correction coefficients based on environmental parameters and additional NO_2_ readings from redundant sensors. Additionally, the calibration model explores extended input parameters, including differentials of environmental variables and historical time series data from the primary sensor, proving their significance. Global data scaling acts as the final step, enhancing scatter plot symmetry and consequent reduction in prediction errors for the calibrated sensor.

Our technique was applied and validated on a monitoring platform developed at Gdansk University of Technology, Poland, comprising primary and secondary NO_2_ detectors, environmental sensors, and custom-designed electronic systems for data transmission and monitoring protocols. The validation involved data from public monitoring stations in Gdansk, Poland. Extensive comparative experiments across diverse calibration model configurations underscored the importance of the integrated algorithmic components. The most comprehensive setup, encompassing all correction mechanisms, demonstrated exceptional reliability, achieving a correlation coefficient of 0.95 between reference and corrected sensor data, with an RMSE below 2.4 µg/m^3^ (an average relative RMS error of just eleven percent). This high efficacy underscores the practical viability of low-cost NO_2_ monitoring.

Future endeavors will focus on refining the precision of calibrated low-cost NO_2_ monitoring. One avenue involves integrating supplementary gas detectors like SO_2_, CO, and O_3_ into the measurement platform. This addition aims to leverage their readings as supplemental data sources to further refine the calibration model, particularly regarding cross-sensitivity considerations. Additionally, exploring advanced machine learning methodologies, including convolutional neural networks (CNNs) and recurrent neural networks (RNNs), is on the agenda. RNNs, adept at managing time series of varying lengths, may specifically enhance monitoring reliability by harnessing such data.

## Data Availability

The datasets used and/or analyzed during the current study available from the corresponding author on reasonable request.
